# Hands to Hexapods, Wearable User Interface Design for Specifying Leg Placement for Legged Robots

**DOI:** 10.3389/frobt.2022.852270

**Published:** 2022-04-14

**Authors:** Jianfeng Zhou, Quan Nguyen, Sanjana Kamath, Yaneev Hacohen, Chunchu Zhu, Michael J. Fu, Kathryn A. Daltorio

**Affiliations:** ^1^ Department of Mechanical and Aerospace Engineering, Case Western Reserve University, Cleveland, OH, United States; ^2^ Department of Electrical, Computer, and Systems Engineering, Case Western Reserve University, Cleveland, OH, United States; ^3^ Department of Physical Medicine and Rehabilitation at the MetroHealth System, Cleveland, OH, United States; ^4^ Department of Computer and Data Sciences, Case Western Reserve University, Cleveland, OH, United States; ^5^ Department of Biomedical Engineering, Case Western Reserve University, Cleveland, OH, United States; ^6^ Cleveland FES Center, Louis Stokes Cleveland Department of Veterans Affairs Medical Center, Cleveland, OH, United States

**Keywords:** human-robot interaction, legged robots, data glove, teleoperation, integrated planning and control, obstacle avoidance

## Abstract

Specifying leg placement is a key element for legged robot control, however current methods for specifying individual leg motions with human-robot interfaces require mental concentration and the use of both arm muscles. In this paper, a new control interface is discussed to specify leg placement for hexapod robot by using finger motions. Two mapping methods are proposed and tested with lab staff, Joint Angle Mapping (JAM) and Tip Position Mapping (TPM). The TPM method was shown to be more efficient. Then a manual controlled gait based on TPM is compared with fixed gait and camera-based autonomous gait in a Webots simulation to test the obstacle avoidance performance on 2D terrain. Number of Contacts (NOC) for each gait are recorded during the tests. The results show that both the camera-based autonomous gait and the TPM are effective methods in adjusting step size to avoid obstacles. In high obstacle density environments, TPM reduces the number of contacts to 25% of the fixed gaits, which is even better than some of the autonomous gaits with longer step size. This shows that TPM has potential in environments and situations where autonomous footfall planning fails or is unavailable. In future work, this approach can be improved by combining with haptic feedback, additional degrees of freedom and artificial intelligence.

## 1 Introduction

**FIGURE 1:**
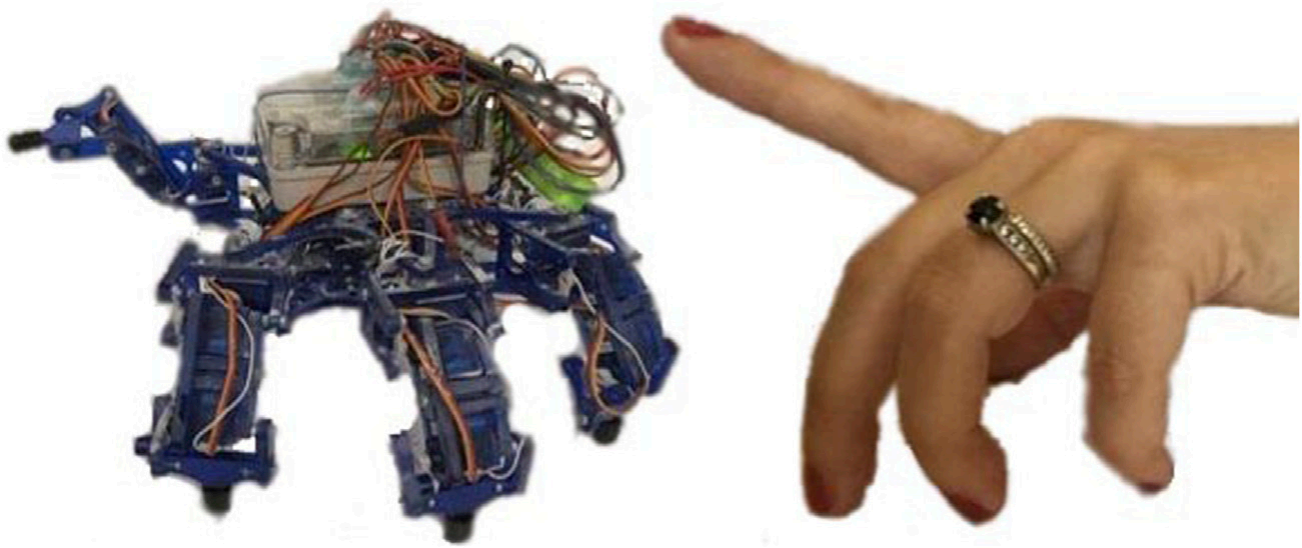
Hexapod robots (left) and human hands (right) have similarities that can be convenient for user interfaces. For example, a user might want to use a single finger to lift a single leg of this crab-like hexapod (Bjelonic et al., 2016; Franchi et al., 2012; Carpentier and Mansard, 2018; Grezmak et al., 2021; Graf et al., 2019; Graf et al., 2021).

Many robots are designed for environments that are too dangerous or remote for human workers. However, the more complex and dangerous the task is, the more likely human oversight will be needed to handle unexpected situations. Thus, while many legged robots are ideal mechanically for mobility on irregular terrain (Hunt et al., 2011; Taylor et al., 2008; Sartoretti et al., 2018), controlling legs with autonomous gaits can limit adaptability potential of these robots. While autonomous gaits are improving due to artificial intelligence (Sartoretti et al., 2019) and bio-inspiration (Hunt et al., 2017, 2014) sometimes the human operator needs to intervene (Wang et al., 2015; Prasanga et al., 2019), a process which can be challenging and tedious. Naive or minimally-trained operators can be safer and more effective robot users with intuitive user control interfaces that allow them to specify individual leg motions (Elliott et al., 2016). Here, our goal is to show that specifying leg motions using finger motions on one hand is feasible, as shown in Figure 1.

This will be especially important for human-robot teams. For example, if the human operator is an expert in a particular environment, direct teleoperation (He et al., 2017) will be needed if the human can recognize obstacles better than the robot can. However, without an intuitive user interface, it can be wearying (Huang et al., 2021) to control each joint to walk for long distances.

This paper introduces a hand-to-hexapod control interface (HHCI) with aims to reduce user effort, while specifying leg positions. The user’s hand motions will be tracked with a wearable device. The advantage of wearable hardware as opposed to soft gloves or camera tracking of the hand is that, later, it can be augmented with haptic feedback.

**FIGURE 2:**
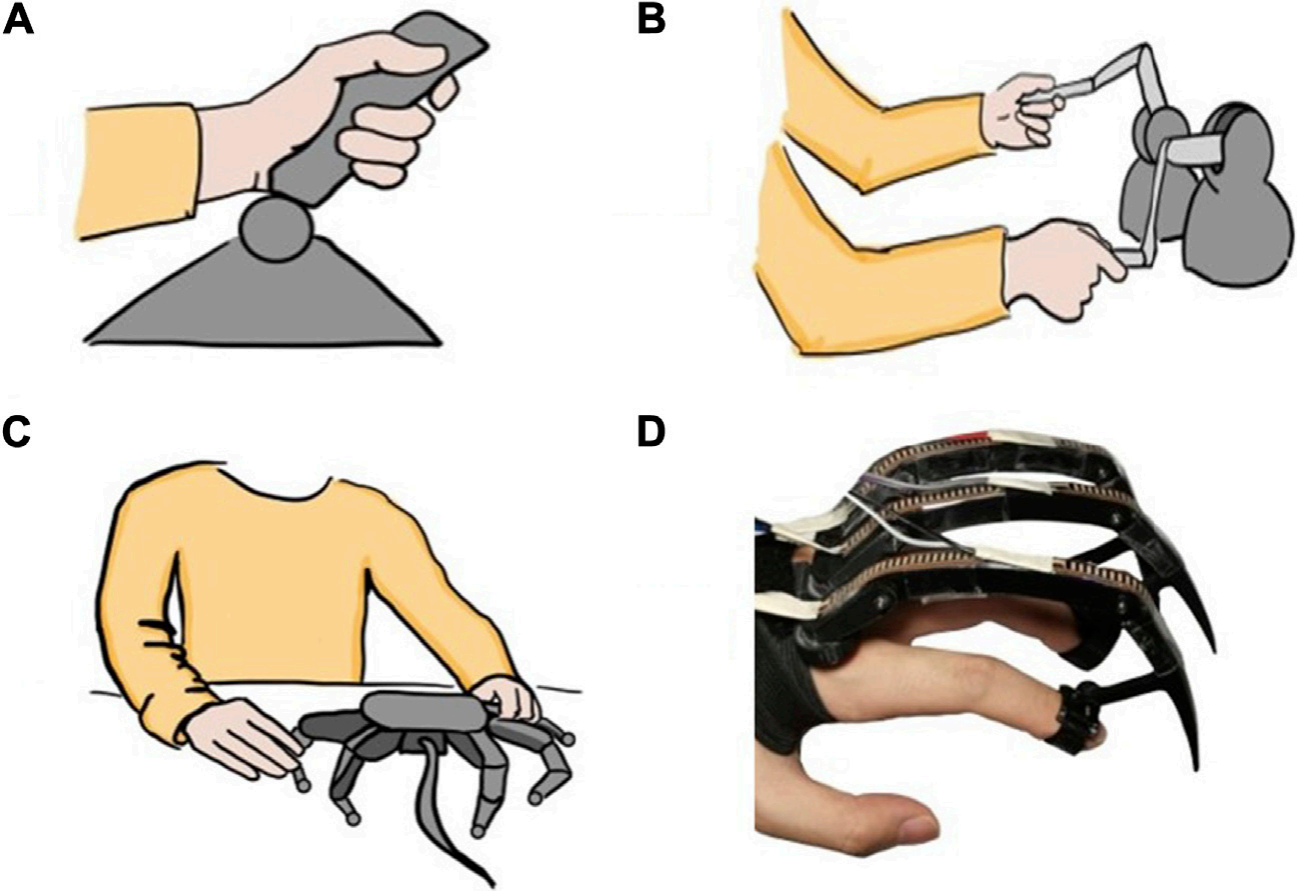
Different control interfaces for hexapod locomotion control **(A)** A joystick control interface (Kurisu, 2011) is easy to use for indicating general direction of walking, but would be frustrating if trying to have robot step in particular spots **(B)** Haptic device interfaces (Huang et al., 2021) provide helpful feedback to the user, but require large arm movements of both arms **(C)** A scaled model (Mae et al., 2017) enables detailed control of each joint but would be slow and tiring to move each joint **(D)** Our proposed HHCI enables precise placement of feet with small finger motions on one hand.

This is novel compared to current methods for controlling hexapod robots. For hexapod robots (Bjelonic et al., 2016; Franchi et al., 2012; Carpentier and Mansard, 2018; Grezmak et al., 2021; Graf et al., 2019; Graf et al., 2021), a simple control interface like joystick or arrows on a keyboard (Kurisu, 2011) is easy and intuitive for the operator to control the locomotion velocity, direction, attitude or even step size, as pictured in Figure 2A. However, both these control interfaces can only control the robot to do pre-programmed motions. In operation, the pre-programmed commands may be too general. For example, detailed control may be desired to step on a specific spot, use a foot to shift objects to make a path in a cluttered area, or brace with a non-end-effector (e.g., a “knee”) in a confined space. A complicated control interface, like a scaled model of the robot (Mae et al., 2017), provides complete and direct control over the robot legs, as pictured in Figure 2B. The disadvantage is that it requires excessive attention and operation from the operators. During locomotion control, the operators have to move their hands to adjust different scale model leg joints, while at the same time analyzing the environment. This can distract the operators, increase the mental demand and decrease the control efficiency.

Haptics are promising because users can feel the terrain through the force feedback and adjust the gait, which aids balance when walking in 3D terrain. Current haptic devices being applied in teleoperation of legged robots include Phantom Omni (DelftHapticsLab, 1994; Barros et al., 2015; Huang et al., 2019) and Touch 3D stylus (Hoshino et al., 2018; Protocom, 2022). With one device paired with one tripod gait group, the operators are able to control the movement of robot legs (Huang et al., 2021), as shown in Figure 2C. Since the operators grasp these devices like a pen, both gross and fine motor of each arm can be performed and translated to the relevant tripod. However, the movement of arms can increase the physical activity and effort. A second disadvantage is that the operator must devote both arms to the full operation of the robot, which may limit multiple parallel operations. Our proposed HHCI could someday incorporate haptic feedback in a one-hand device, leaving the second arm free.

A specific kind of glove is needed to map finger motions directly to leg motions that is different from other data gloves that have been developed for robotic control. In the literature, there are two methods for capturing finger motion with gloves. One is detecting the operators finger flexion (the joint angles of the hand), which is typically done with soft gloves (O’Flynn et al., 2013; Kessler et al., 1995) that characterise gestures. However, incorporating rigid elements can make these measurements more precise (Chen et al., 2020). The difference in our work is that rather than characterizing gestures that correspond to pre-programmed actions (Chen et al., 2015; Liu et al., 2016), the goal is to directly control individual legs and determine whether that can be sufficient for tasks like obstacle avoidance. The second method is to measure finger tip motions. For example, hardware gloves provide haptic feedback at fingertips in Virtual Reality environments (Gu et al., 2016; Friston et al., 2019). Here, the component being controlled in virtual reality is not a human hand but a robot leg, and our hypothesis is that having a physical leg model attached to the user’s hand is helpful in improving control efficiency.

**FIGURE 3:**
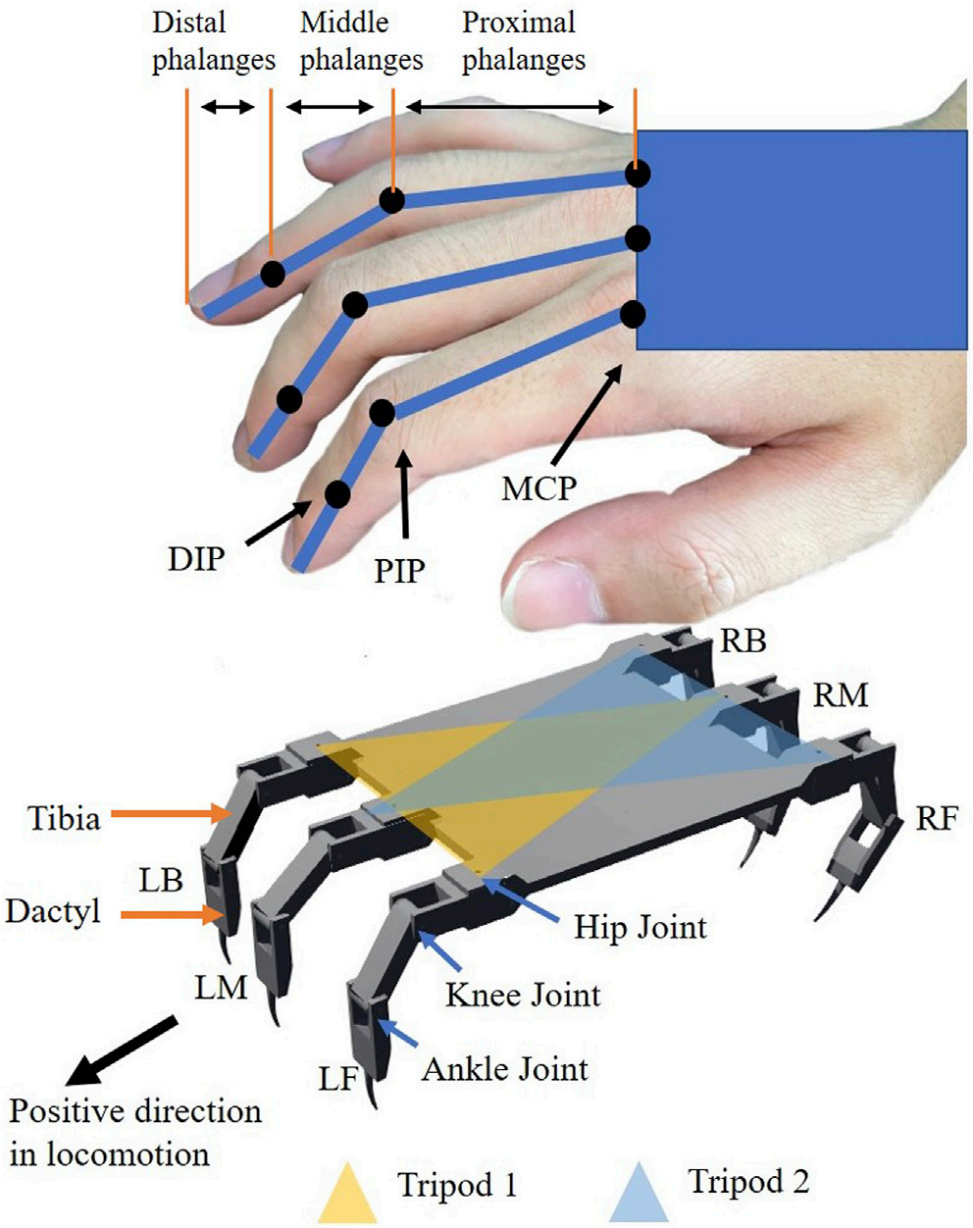
The joints in a hand (above) are mapped with kinematics to control the legs of a hexapod robot (below). Legs are divided into two groups to perform tripod gait in sideways walking. Left-Front leg (LF), Left-Back leg (LB) and Right-Middle leg (RM) are in Tripod 1. Right-Front leg (RF), Right-Back leg (RB) and Left-Middle leg (LM) are in Tripod 2.

In this paper, as shown in Figure 3, a HHCI is proposed to take advantage of the similarities in structure between a human finger and the three degree of freedom (DOF) leg of a hexapod robot. Users are able to directly control the leg movements of a hexapod robot with finger movements. Two different gloves with different mapping methods, Joint Angle Mapping (JAM) and Tip Position Mapping (TPM), are tested and compared to find out which mapping method is more efficient in specifying hexapod leg placement. The glove with better efficiency (TPM) is then tested in a task-based simulation. The test environment was built in Webots simulator, in which operators are asked to control the robot to perform a sideways walk and avoid obstacles. Then the results are compared with fixed gaits and camera-based autonomous gaits to evaluate the performance of the HHCI. In the end, the advantages of HHCI are clearest in challenging cluttered environments, where they are comparable to autonomous gaits. Since autonomous gait may fail to traverse many environments, it is important to have user interface alternatives. This work is the first to introduce HHCI, which can be more broadly applied to many legged robot tasks in future to improve robot usability and adaptability.

## 2 Materials and Methods

The goal of our glove is to relate motions of a common hexapod leg to finger motions. A common robot leg design has three joints (Graf et al., 2019, 2021; Wooden et al., 2010; Boston Dynamics, 2022; Michael, 2012; Hwangbo et al., 2019; Darling, 2015; Sartoretti et al., 2018; Coelho et al., 2021), as shown in Figure 3: a hip joint with vertical axis of rotation, a knee joint, and an ankle joint with parallel axes of rotation. Just like one robot leg has three joints, there are three joints on one finger: the metacarpophalangeal joint (MCP), the proximal interphalangeal joint (PIP) and the distal interphalangeal joint (DIP) (Jones and Lederman, 2006; Palastanga and Soames, 2011; Wheatland et al., 2015). There are three segments on one finger: the proximal phalanges, the middle phalanges, the distal phalanges. For human fingers, the MCP has two DOF. The abduction and adduction movement of MCP corresponds to the movement of the robot’s hip joint. During flexion and extension movement, the motion of the proximal phalanges on human hand is similar to the desired motion of the tibia on the robot. Thus, the flexion and extension movement of MCP corresponds to the movement of the robot’s knee joint. The robot the has one additional flexion joint, the ankle joint, which will be controlled by the human PIP and DIP movements. The limited flexibility of DIP and the coupling with PIP makes it almost impossible for DIP to control the ankle joint without PIP. With PIP occupied, MCP is the only joint which can correspond to the knee joint.

For this work, we will focus on planar sideways walking, so that we only need to track two finger joints. Prior research has evidenced that sideways walking is faster and more efficient than forward walking for a hexapod robot (Yang Chen et al., 2021). Furthermore, compared to sideways walking, forward walking requires frequent movement of hip joints, which corresponds to the abduction and adduction movement of MCP. However, the abduction and adduction angles of MCP are limited, and frequent abduction and adduction movement can cause discomfort to the operator, leading to a faster muscle fatigue. In contrast, sideways walking can make full use of the flexibility of fingers in flexion and extension without making the operators feel uncomfortable. Therefore, the glove only detects flexion and extension movements of the fingers, leaving abduction and adduction tracking for future work.

**FIGURE 4:**
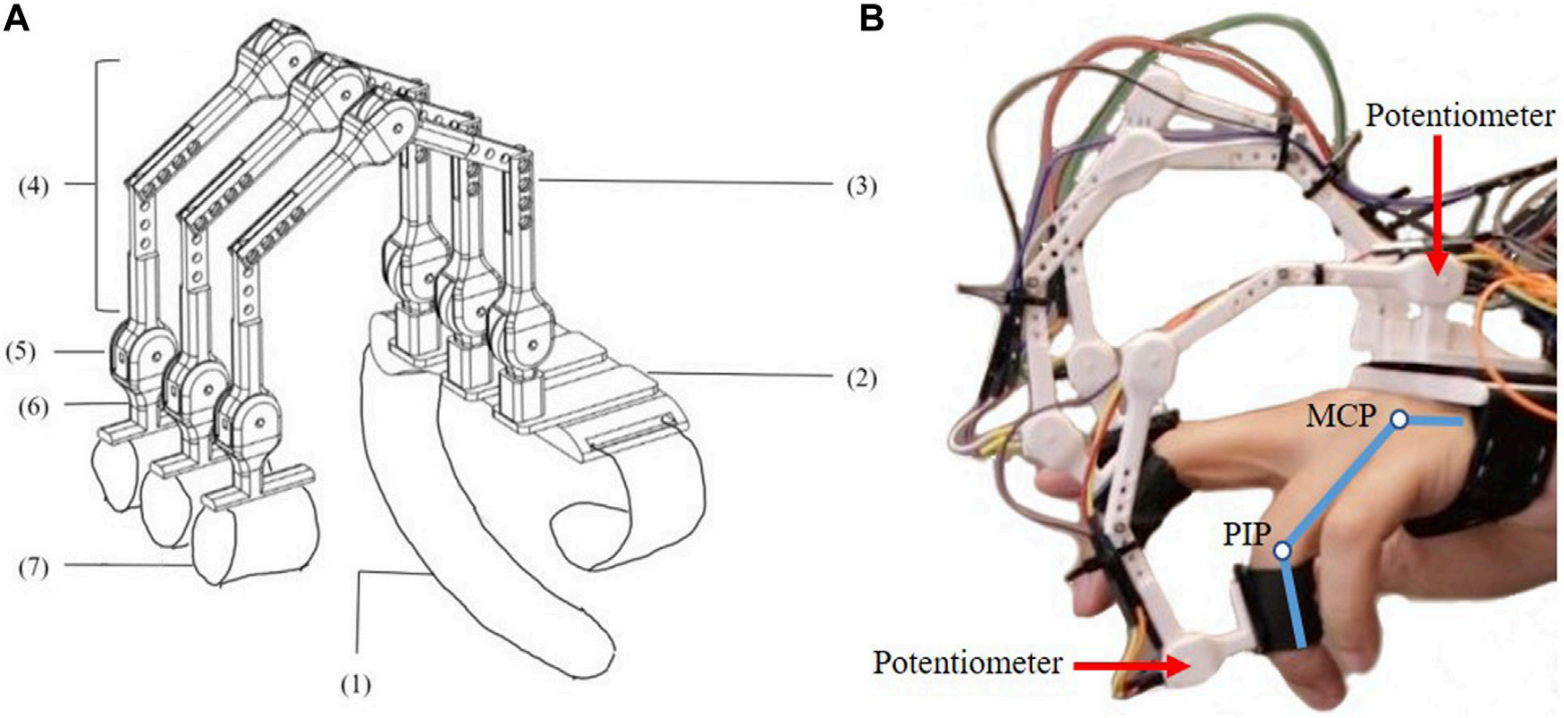
Glove one is designed for Joint Angle Mapping (JAM) **(A)** The design consists of (1) Elastic band (2) 3D printed base. (3) 3D printed Finger Segment 1. (4) 3D printed Finger Segment 2. (5) Linear Rotary Potentiometer (PT10MH01-103A2020-S, 10 kΩ, 0.15W) (6) 3D printed finger attachment support (7) Finger Straps **(B)** The user wears the glove to measure MCP and PIP flexion.

**FIGURE 5:**
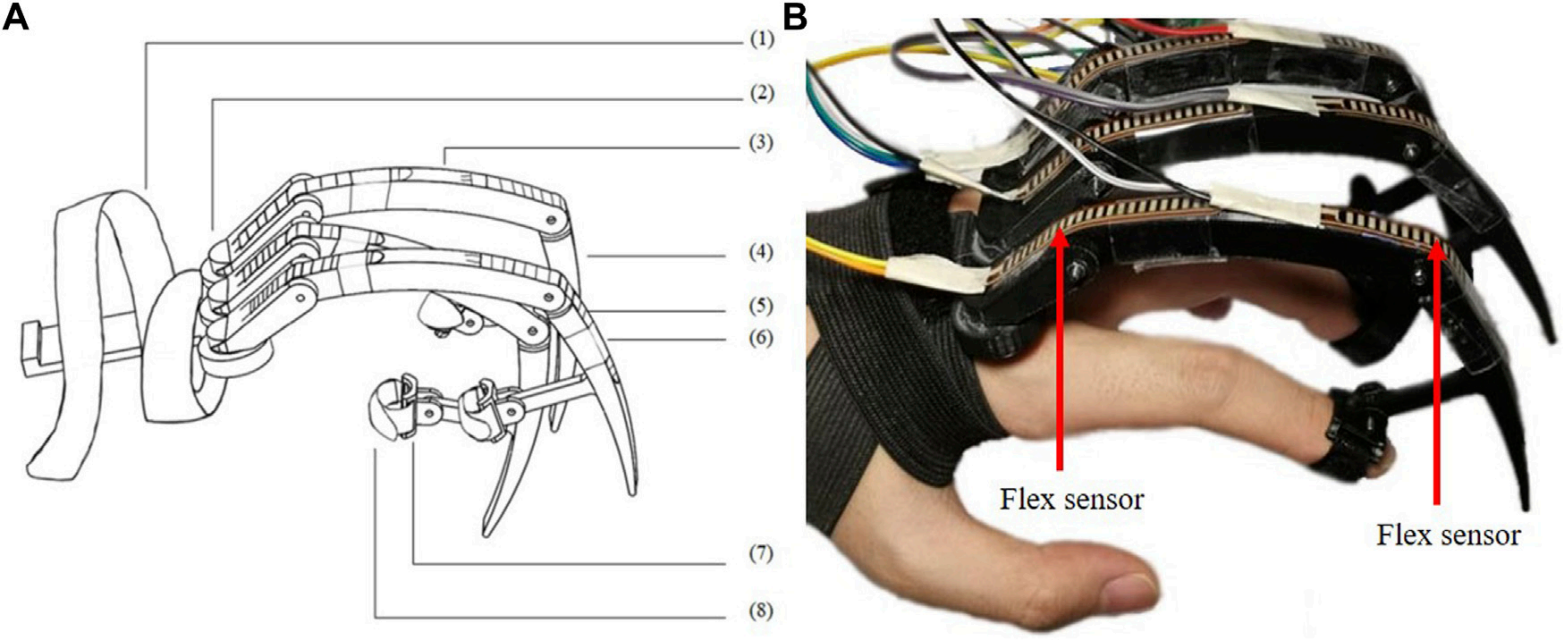
Glove 2 is designed for Tip Position Mapping (TPM) **(A)** The design consists of (1) Elastic knit band with Velcro (2) 3D printed base. (3) 3D printed dactylus 1. (4) 3D printed dactylus 2. (5) Flex sensors (Adafruit Short Flex Sensors 25kΩ - 100 kΩ) (6) Slides (7) 3D printed ring (8) Finger strap **(B)** The user is shown wearing the device such that tip motion is a result of MCP, PIP and DIP flexion.

There are multiple ways to map finger movement to the robot legs to take into account the differences in kinematics between hands and the robot (Friston et al., 2019; Wang et al., 2019; Chen et al., 2015; Napier, 1956; Taylor and Schwarz, 1955; ElKoura and Singh, 2003; Haken et al., 1985). For the crab robot model used in our lab, the dactyl will be 60% longer than the tibia while human hands have variability in the lengths of the three segments with the last two joints being coupled. In this research, two mapping methods are considered. To compare, two gloves are designed, as shown in Figure 4 and Figure 5. Both gloves are fixed on the operator’s hand by an elastic band with Velcro.

### 2.1 Glove 1: JAM Glove

Glove one is for joint angle mapping (JAM). The goal is to use the finger angles to directly set the robot joint angles. The MCP will correspond to the knee joint motion. The ankle joint will correspond to the PIP rather than the DIP motion because although they are coupled, the PIP has better flexibility and larger work space than DIP. Thus, The fingertips of Glove one are fixed on the middle phalanges of the operator through 3D printed rings and finger straps. The movement is detected by potentiometers, whose voltages will be recorded and used to calculate the flexion and extension angles of MCP and PIP through inverse kinematics. In this way, the user is able to control the robot joints by mapping the finger joint angles directly to the robot leg joint angles.

### 2.2 Glove 2: TPM Glove

Glove 2 is for tip position mapping (TPM). Here, the DIP motion is included because total flexion is captured at the finger tip. The finger tips of Glove 2 are fixed on the distal phalanges of the operator through 3D printed rings and finger straps. The movement is detected by flex sensors, whose voltages are recorded and used to calculate the resulting finger tip position through forward kinematics. Inverse kinematics are applied to get the corresponding robot joint angles for the legs. The user is able to control the robot foot tip positions by mapping the finger tip positions directly to the robot foot tip positions.

For the TPM Glove, the user can visualize the robot’s leg by looking at the hardware dactyl attached to the finger, which has the same proportions as the robot’s leg. In contrast, for the JAM Glove, the leg motions correspond more directly to the operator’s finger.

Two quantitative tests are performed to select which type of glove will be used.

### 2.3 Precision Test Set-Up

The precision test is used to check whether the sensor’s value is consistent during repeatable movement. According to tests made by other researchers (Roy et al., 2015; Glauser et al., 2019), a standard deviation and mean error within 10°is precise enough for a glove’s sensor. The test made by Oliver G (Glauser et al., 2019).shows that the ManusVR glove has a mean error of 11.93°and the CyberGlove 10.47°.

During the test, the glove is not worn, but rather the base is fixed on a platform of fixed height. Reference positions A and B are marked on a paper template and the glove fingers are moved to these two marks. At position A, the foot is taped to the mark. At rest, the sensor voltages are sampled 20 times with MATLAB. Then the finger tip is moved to position B, and the sensors are read again. The test is repeated 20 times, recording 400 values for each sensor on each position. the mean and standard deviation of all recordings of each sensor on each position are calculated. A glove with lower standard deviation values and mean error can be considered as the glove which is more stable and precise in recording values of repeated positions.

### 2.4 Interaction Efficiency Test Set-Up

Next, it is important to compare performance when a human user is added to the control loop. This is different from the previous precision test, because the human user can adjust the position of their finger to achieve a desired result in real time (Yanco et al., 2015).

**FIGURE 6:**
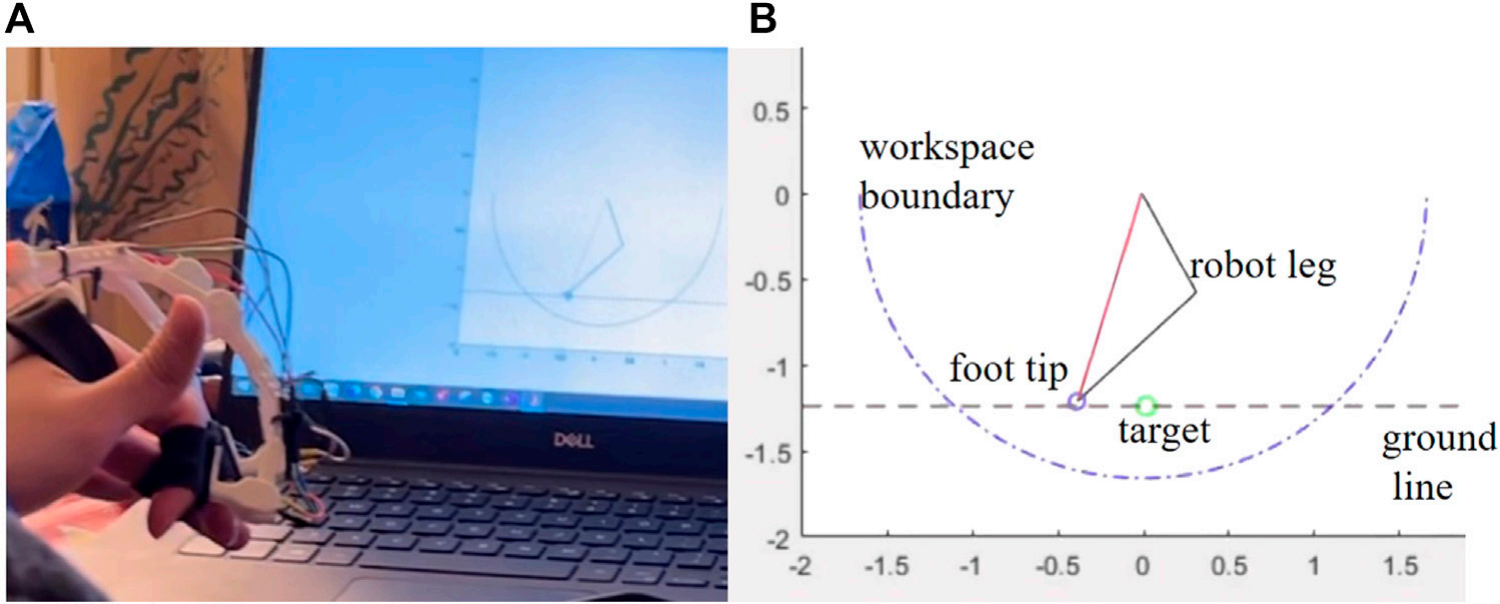
**(A)** An operator controls a simulated robot leg to reach a target position on the simulated ground **(B)** Simulation environment for interaction efficiency test.

Here, we will measure how quickly and accurately the user can get a single simulated leg into position. This test is a simplified simulation of sideways walking control for hexapod robot, in which specified leg placement is required. 15 lab staff were invited to the test, using their index fingers to control a simulated robot leg with both gloves to reach a certain target position on the simulated ground, shown as Figure 6. All the staff are new to the gloves and have never been trained before. One full test of each glove consists of a sequence of 15 randomly generated target positions. Once the robot foot tip crosses the ground line or touches the target, the trial will be ended and the target position will be refreshed.

The efficiency is quantified in two dimensions. The first dimension is the time spent. The time for each trial reflects the effort and frustration during operation. The less time spent means the less effort required and the less frustration during operation, in other words, it is easy to operate. The second dimension is the distance between the target and the final foot tip position. Errors in distance reflect if the user controls the foot to impact the ground earlier or later than the desired position, which reflect the effectiveness of performance. A small distance means the user can perform effectively and reduce the risk of touching obstacles by mistake when specifying leg placement. The results are filtered out if the distance is larger than 5cm, which means the user fails to reach the target or impacts the ground too early before reaching the target. If a user fails more than five times on either glove, all the data on both gloves from that user will be excluded. There are ten users failing less than five times, whose average time and average distance are recorded.

### 2.5 Comparing JAM and TPM Gloves

#### 2.5.1 Precision

The result of precision test for index finger is shown in Table 1.

**TABLE 1:** Results of precision test for index finger.

Reliability Test		Glove 1		Glove 2	
Position	Sensor	Std. Dev.(°)	Mean Error (°)	Std. Dev.(°)	Mean Error (°)
A	Sensor 1	0.4	0.3	2.4	2.0
	Sensor 2	0.7	0.6	8.6	6.8
B	Sensor 1	0.5	0.4	1.8	1.5
	Sensor 2	0.5	0.4	5.7	5.0

The potentiometers of Glove one have lower standard deviation values and lower mean errors than the flex sensors of Glove 2, which means that Glove 1 may be more precise and reliable. However, the flex sensors are lighter and easier to integrate into wearable devices in field applications. Therefore we performed other tests to show that the precision of Glove 2 is sufficient for this application.

#### 2.5.2 Interaction Efficiency

**FIGURE 7:**
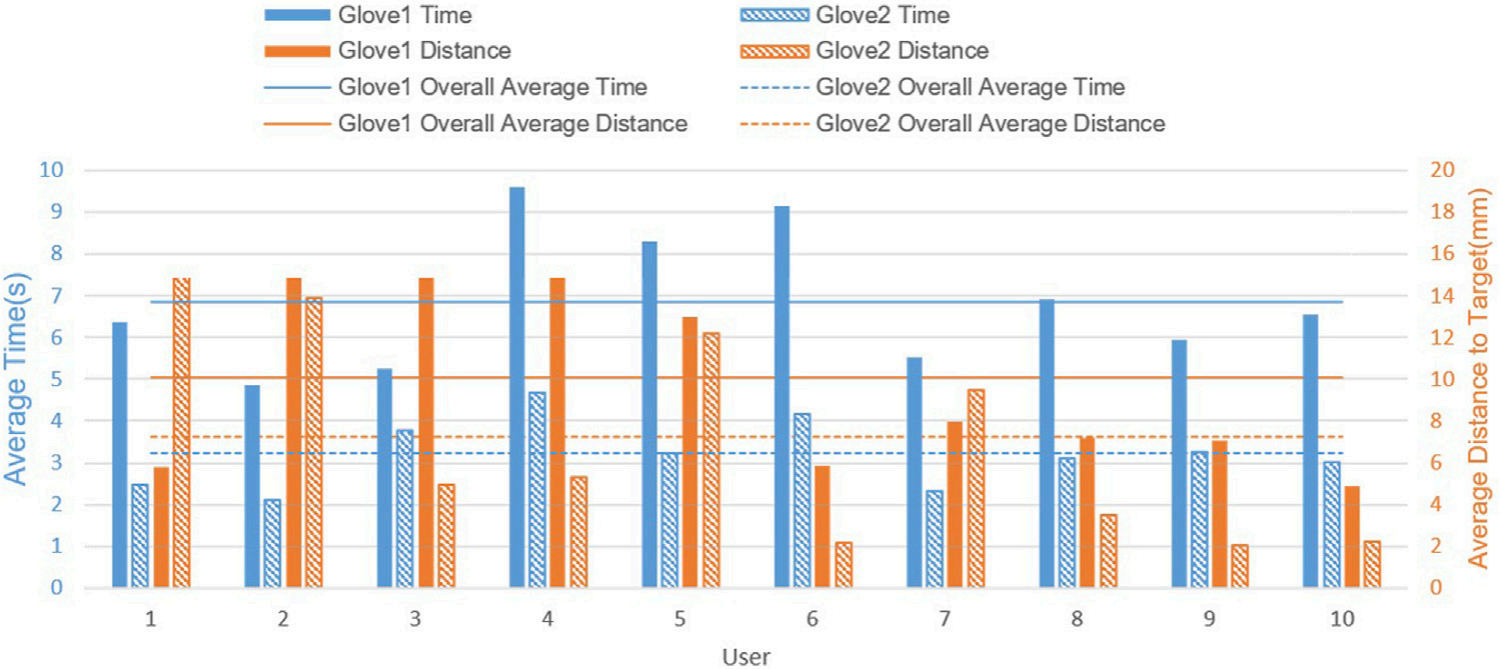
Result of interaction efficiency test.

Glove 2 was better than Glove one in both time and distance, suggesting that TPM is overall more intuitive for users. As shown in Figure 7, all users consume less time when operating Glove 2 (TPM). This suggests that TPM is more promising in reducing mental demand and effort. Most of the users, except two of them, can get closer to the goal with Glove 2. This suggests that for most users, TPM is better than JAM for performance overall, despite the fact that the sensors on Glove 2 are less precise. In summary, Glove 2 (TPM) is more user-friendly and effective in specifying leg placement for a hexapod robot.

### 2.6 TPM Evaluation Methods

#### 2.6.1 Task Set-Up

**FIGURE 8:**
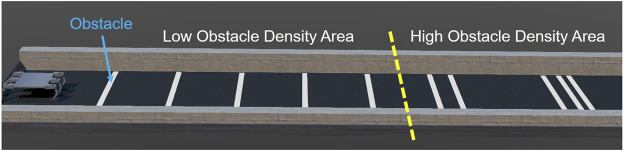
Simulation environment set-up.

**FIGURE 9:**
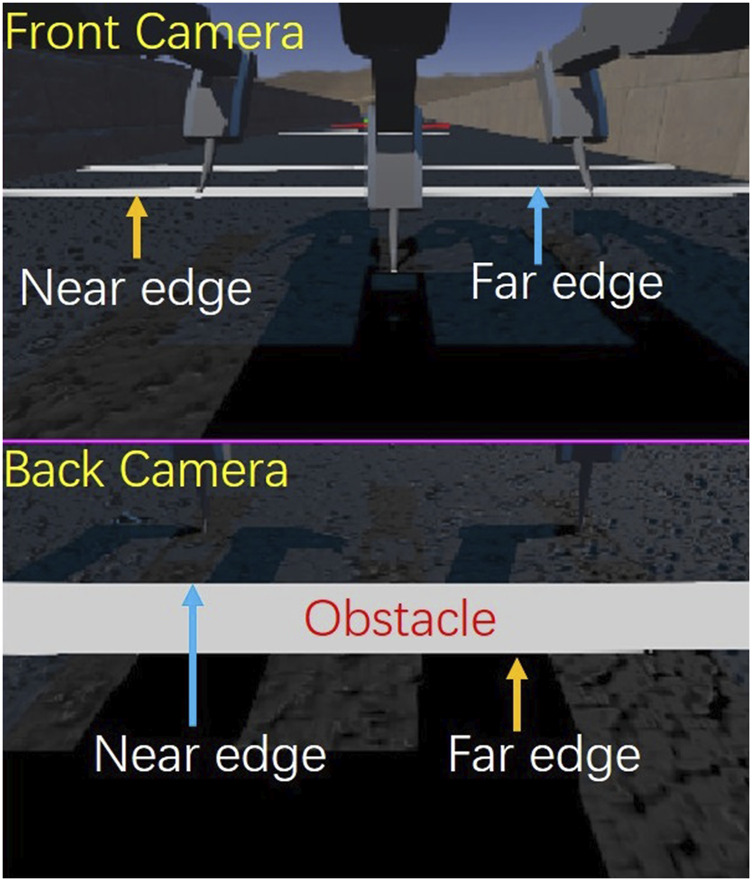
The front and back camera views of the robot.

To verify the HHCI, a task is carried out in Webots simulation, as shown in Figure 8. In the task, the most experienced operator needs to control the robot walking through a straight lane with white stripes as obstacles. Each obstacle is 5 cm in length. The lane is divided in 2 sections, with five obstacles to avoid in each. The first part has a lower obstacle density while the next part has a higher obstacle density. The goal is to avoid stepping on the obstacles during locomotion control. Every contact with the obstacles is counted. During manual locomotion control, only the camera display windows are shown to the operator, as show in Figure 9, so that the operator can observe the environment and the robot movement in a first-person view instead of a global perspective.

#### 2.6.2 HHCI for Tripod Gait

Here our HHCI enables manual control of a tripod gait, as shown in Figure 3. The movement of LF is controlled by the movement of index finger while the other two legs in Tripod1 (LB and RM) follow the movement of LF. The LM is controlled by middle finger while the other two legs in Tripod2 (RF and RB) follow the movement of LM to keep body balance and avoid slipping on the ground. All the foot tips in the same tripod share the same vertical position and horizontal velocity. The operator only needs to use two fingers to control the robot locomotion. Thus, the operator can focus on locomotion control and environment analysis without being distracted by finger coordination and robot balance. According to the interaction efficiency test, TPM has better efficiency. So, TPM glove is used by an experienced operator to control the locomotion of the hexapod robot during the test. Thus, the third and fourth fingers of the user’s hand are not used here. In future work, the five fingers of the hand could be used in different configurations to control the six legs of the robot in different modes.

Fingertip positions (*x*_*i*_, *y*_*i*_) (*i* = 1, 2), the corresponding foot tip positions (*X*_*i*_, *Y*_*i*_) are defined as the following.

$$\left[\begin{array}{cc} X_{1} & Y_{1}\\ X_{2} & Y_{2} \end{array}\right]=k\left[\begin{array}{cc} x_{1} & y_{1}\\ x_{2} & y_{2} \end{array}\right]+\left[\begin{array}{cc} \delta_{x1} & \delta_{y1}\\ \delta_{x2} & \delta_{y2} \end{array}\right]$$ (1)

k is the scaling ratio, a positive and real constant depending on the glove’s finger size. k equals to the ratio between the robot leg length and the glove’s finger length. (*δ*_*xi*_, *δ*_*yi*_) form position adjustment vectors to counteract the displacement between the glove and the operators hand.

The inverse kinematic equations for left side legs are

$$\alpha_{i}=\frac{\pi }{2}-\arctan\frac{X_{i}}{Y_{i}}-\arccos\left(\frac{X_{i}^{2}+Y_{i}^{2}+L_{1}^{2}-L_{2}^{2}}{2L_{1}\sqrt{X_{i}^{2}+Y_{i}^{2}}}\right)\;\left(i=1,2\right)$$ (2)

$$\beta_{i}=\pi -\arccos\left(\frac{L_{1}^{2}+L_{2}^{2}-X_{i}^{2}-Y_{i}^{2}}{2L_{1}L_{2}}\right)\;\left(i=1,2\right)$$ (3)

*α*_*i*_ are the angles of the knee joints. *β*_*i*_ are the angles of the ankle joints (*X*_*i*_, *Y*_*i*_) are the foot tip positions relative to the knee joints. *L*_1_ is the length of robot tibia. *L*_2_ is the length of robot dactyl. Since the right-side legs are bending opposite to the left legs, foot tip positions for the right legs relative to the knee joints in inverse kinematic equations should be (−*X*_*i*_, *Y*_*i*_) to keep the right legs moving in the same direction and velocity as the left legs.

During locomotion control, the operator will first predict the obstacle’s distance through the obstacle’s position in the camera view. One step is divided into two phases, stance and swing. Swing distance is the horizontal distance that the foot tip passes relative to the robot body when it swings in the air. Stance distance is the horizontal distance that the foot tip passes relative to the robot body when it contacts the ground. The step size of the robot is equal to the stance distance. The operator will adjust the swing distance and stance distance to avoid stepping on the obstacle. The operator will decrease the swing distance and put the foot tip to a closer position if the obstacle’s near edge is close to the predicted footfall position. If the obstacle is close to the robot and the far edge is close to the predicted footfall position, the operator will take a larger step to go over the obstacle.

#### 2.6.3 Comparative Experiment Set-Up

A group of fixed gaits is set as for baseline comparison in the experiment groups. Three different step lengths for fixed gaits are tested. For fixed gait, the larger the step length is, the less chance it will have to contact the obstacles because the total contact with ground is reduced. The fixed gaits step lengths are set to be 10 cm, 15 cm and 20 cm to reduce the contact as much as possible. To make sure results are robust to initial conditions, the initial distance from the robot center to the first obstacle’s near edge is sampled randomly from 27.5 cm to 57.5 cm for each step length.

To further compare the obstacle avoidance, a camera-based autonomous gait is designed (Jouaiti and Henaff, 2018; Lee et al., 2017; Sun et al., 2017; Shaw et al., 2019). The input visual information is exactly the same as the camera view provided to the operator. To make the obstacle detection mechanism similar to the human operator, only one camera per side is used to detect the obstacles distance, rather than doing stereo visual depth perception (Howard, 2008). When the obstacle is recognized, its near edge and far edge will be located on the camera image, as show in Figure 9. The vertical pixel position on the image has a corresponding angle of view. Using the view angle, camera angle and the height of the robot, the obstacle distance can be detected.

$$X_{o}=\left(H_{r}+Y_{c}\right)\tan\left(\Psi -\arctan\frac{\left(P_{V}-2P_{o}\right)\tan\frac{\Phi }{2}}{P_{H}}\right)$$ (4)

*X*_*o*_ is the horizontal distance between the obstacle and the center of the robot’s body. *H*_*r*_ is the robot body height. *Y*_*c*_ is the vertical position of the camera in the robot’s body frame. *Ψ* is the pitch angle of camera. *P*_*V*_ is the camera’s maximum pixel number in the vertical direction. *P*_*H*_ is the camera’s maximum pixel number in the horizontal direction. *P*_*O*_ is the obstacle’s pixel position in the vertical direction. Φ is the camera’s field of view.

The strategy of the autonomous gait is modeled after the manual control strategy. When there is no obstacle in front of the legs, the robot will take steps of fixed swing distance and fixed stance distance. When obstacles are detected in front of the robot leg, the robot will predict the obstacle’s position relative to the body center when the swinging foot contacts the ground. The swing distance will be changed to avoid stepping on the obstacles, mimicking strategy in manual control. The swing distance is determined by the predicted obstacle distance. As shown in Figure 11 and Figure 12, if the near edge of the obstacle is close to the original contact position, the robot will decrease its swing distance from *S*_0_ to *S*_1_ to take a smaller step. Determined by the obstacle distance, *S*_1_ is smaller than the obstacle distance to keep a safe distance (1 cm 3 cm) from the obstacle. The subsequent step’s support polygon will be shifted backward relative to the body. The robot will go over the obstacle in the next step. If the far edge of the obstacle is close to the original contact position, the robot will increase the swing distance from *S*_0_ to *S*_2_ to go over the obstacle. The subsequent step’s support polygon will be shifted forward relative to the body. *S*_2_ is larger than the obstacle distance to keep a safe distance from the obstacle. To keep the velocity constant and avoid slipping, the robot only adjusts the swing distance without changing its stance distance (*S*_0_). In other words, the step length of the robot is constant. In the experiment, two different stance distance (*S*_0_) for camera-based autonomous gait are tested. Due to the work space of the robot, the step lengths for autonomous gait are set to be 8 cm and 10 cm while the maximum swing range for the foot tip is ±10 cm. The trajectories of foot tips and obstacles relative to robot body in tripod one during camera autonomous gait are shown in Figure 12.

## 3 Results

### 3.1 Comparison of TPM With Autonomous and Fixed Gaits for Obstacle Avoidance

For both Low Obstacle Density Area and High Obstacle Density Area, the fixed gaits have the most Number of Obstacle Contacts (NOC), as shown in Figure 13. The average NOC, marked by the cross marker in the box-plot, decreases when step size is increased. This is expected because the fixed gaits are “blind” to obstacles and larger steps impact the ground less often.

The results of camera-based autonomous gait are much better than the results of fixed gait, especially in the Low Obstacle Density Area. Compared with the 10 cm fixed gait, the average NOC is reduced by 97% in the Low Obstacle Density Area. While ideally, we want to eliminate all obstacle contacts, (NOC = 0), impacts with the ground cause perturbations in pitch angle which can lead to errors in observed obstacle distance, as shown in Figure 12 from 2 to 3s. To avoid contact resulting from distance error, tolerances are added to the autonomous gait, represented by the radius of the circles in Figure 12. The addition of an IMU, vibration dampers or signal filtering would likely help, however not all autonomous gaits will have these (Avram et al., 2015).

The performance of autonomous gait in the High Obstacle Density Area is not as good as that in Low Obstacle Density Area. Take 10 cm camera-based autonomous gait as an example, the average of NOC increases to three while the maximum NOC increases to 6. The increase in NOC are mainly caused by the misjudgment when there are multiple obstacles in one camera view. The controller is only designed to detect the distance of the nearest obstacle, which leads to possible contact with the following obstacles. While a more complex autonomous gait could be written to have additional layers of control to handle these situations, testing each possible obstacle combination case can be time-consuming.

The results of TPM HHCI gait shows that it is an effective way to avoid obstacles. In the Low Obstacle Density Area, the NOC ranges from 3 to 5, which is much better than blind walking but not as good as camera-based autonomous gaits. In the High Obstacle Density Area, the NOC ranges from 2 to 3, which is close to or even better than the results of camera-based autonomous gait.

## 4 Discussion

### 4.1 Conclusions

**FIGURE 10:**
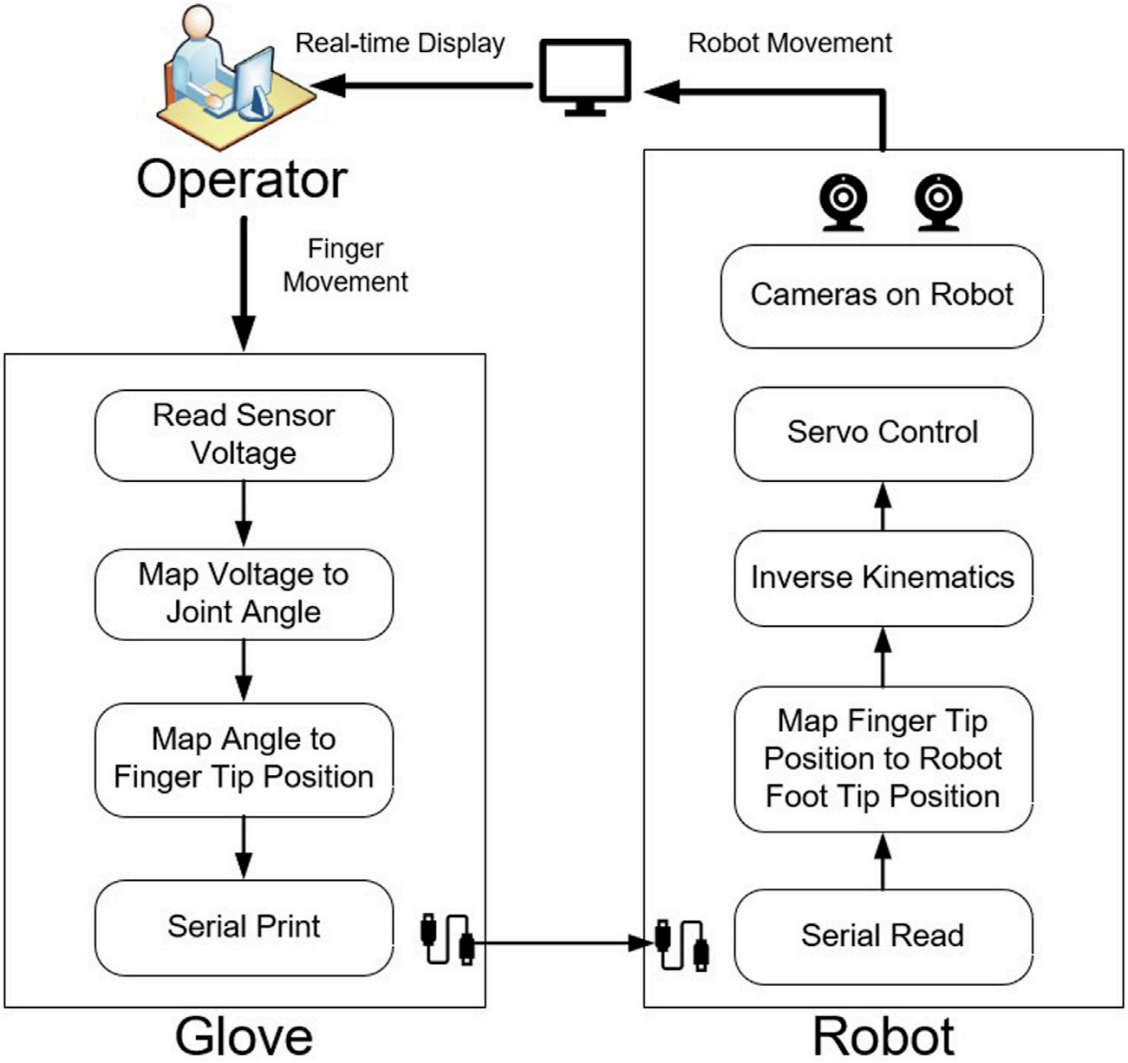
The work flow of the TPM glove control interface.

**FIGURE 11:**
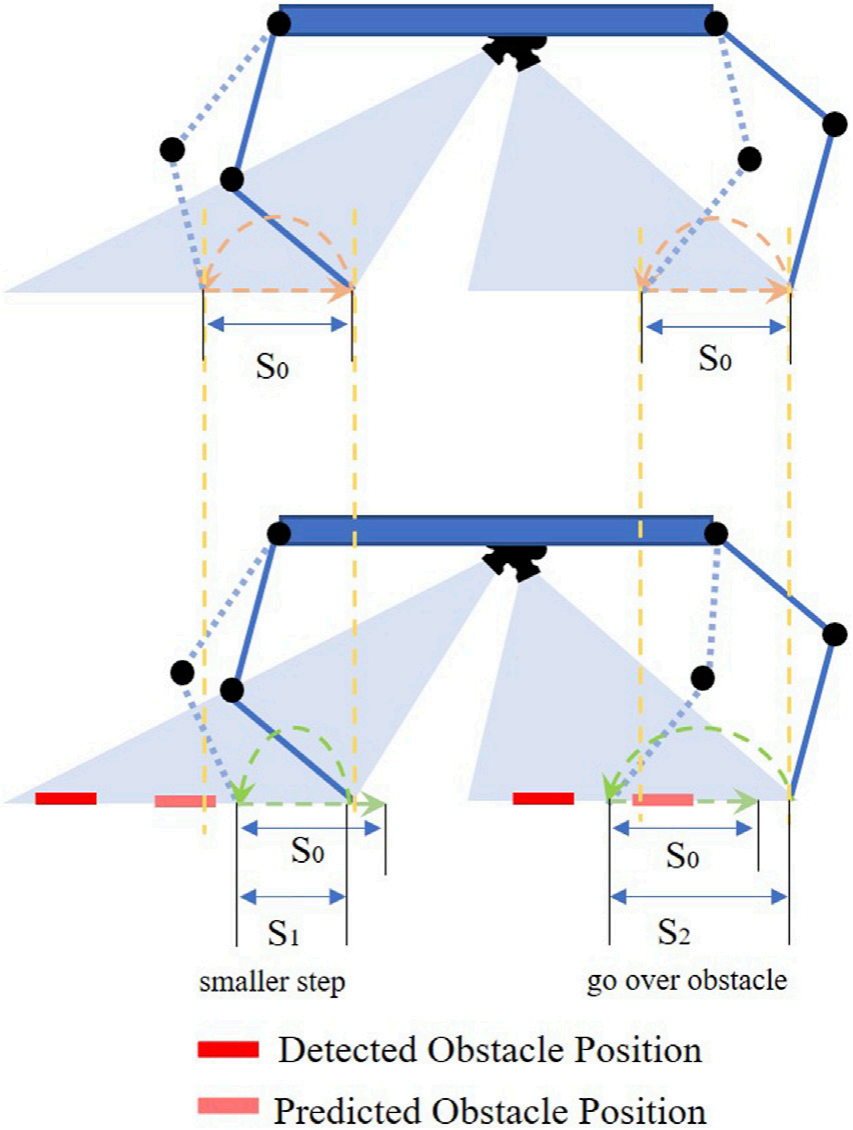
In the camera-based autonomous obstacle avoidance gait, the computer modifies the default swing distance *S*_0_ to a smaller swing distance *S*_1_ or larger swing distance *S*_2_ as needed to avoid obstacles shown in the bottom image. The stance distance (step length) is always the same as the default stance distance *S*_0_. The detected obstacle position is the observed position at the beginning of the swing phase, the predicted obstacle position is where the obstacle will be relative to the robot at the end of the swing phase.

**FIGURE 12:**
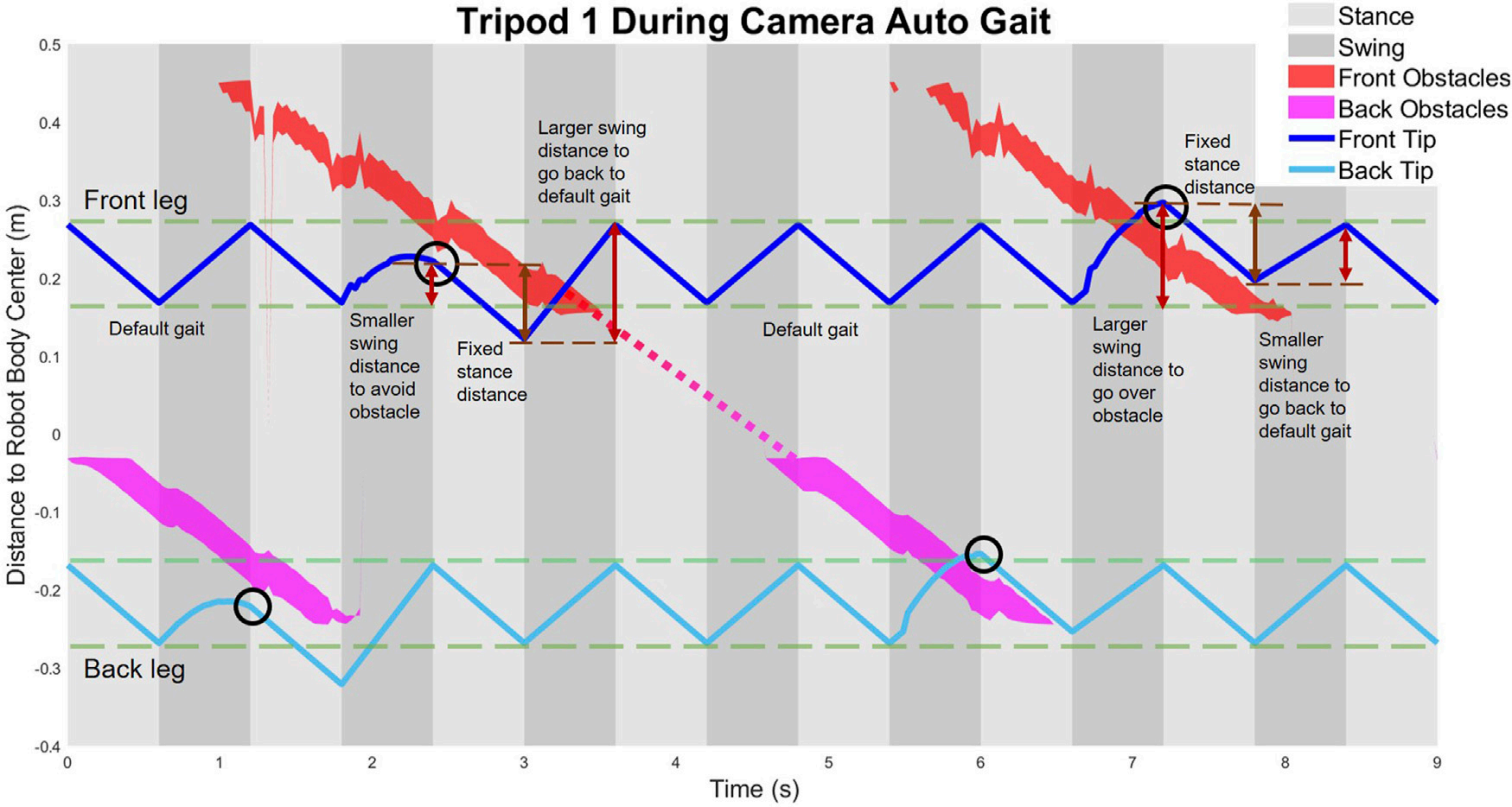
Trajectories of foot tips relative to the robot body in tripod one during camera autonomous gait.

**FIGURE 13:**
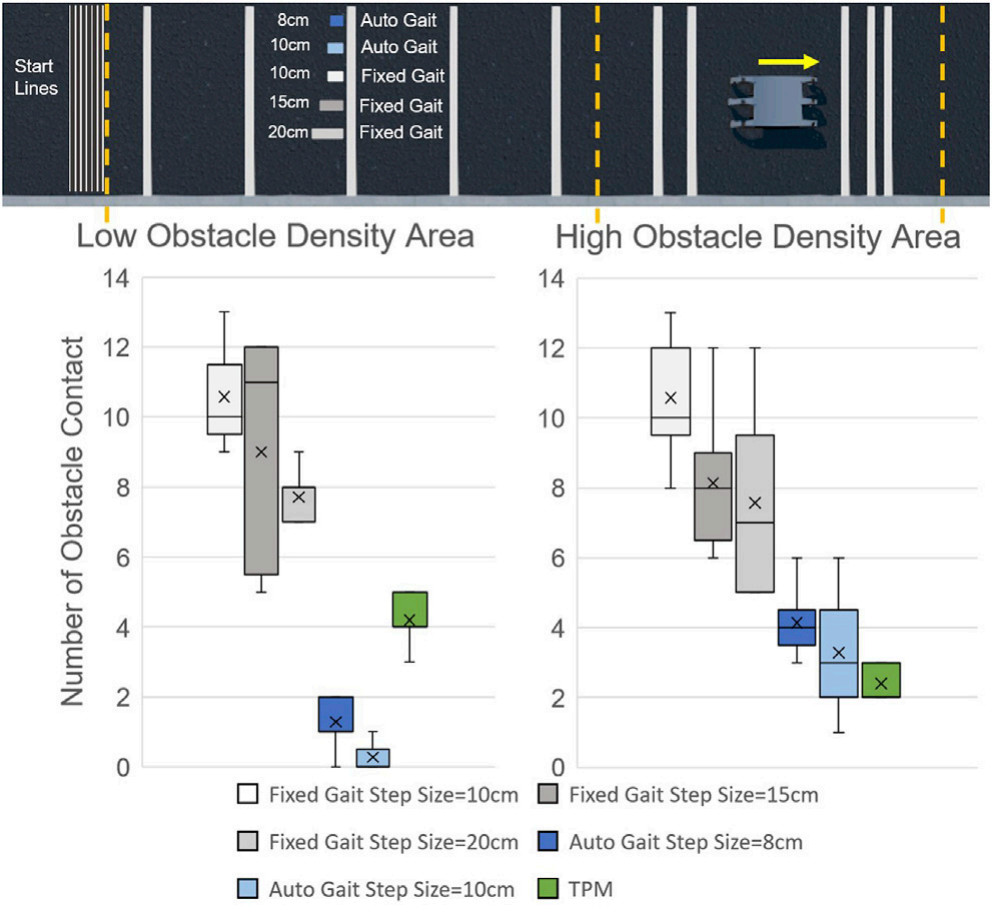
Result of different gaits.

This paper introduces a new control interface for hexapod robots using hand-to-robot mapping to specify leg placement (Figure 1). A simplified real time simulation is built in MATLAB and two control gloves are designed (Figure 2, Figure 3, Figure 4). 15 lab staff are tested with both gloves to determine which kind of mapping method is more intuitive (Figure 5). Glove 2, the tip position mapping glove, is more intuitive in specifying leg placement (Figure 6). To show that this interface can allow users to specify foot positions, a simulation is set up in Webots (Figure 7), in which robots need to use sideways walking (Figure 8) to walk along a straight lane while avoiding bar-like obstacles. In the simulation, manual gait is tested with Glove 2 (Figure 10, Figure 9). To show the worst case scenario, gaits with fixed step size are shown for comparison. A camera-based autonomous gait is designed as to show the minimum computer controlled results (Figure 11, Figure 12). The result (Figure 13) shows that both the autonomous gait and the manual control are effective ways to adjust step size to avoid obstacles. Manual control has advantages over camera-based autonomous gait when there are multiple obstacles on one side (High Obstacle Density Area in Figure 13). This demonstrates that in a situation in which there is no autonomous gait available, a manual control scheme is likely to be comparably accurate in the sagittal plane.

### 4.2 When to Use HHCI

The difference in manual control performance between the Low Obstacle Density Area and the High Obstacle Density Area (Figure 13) mainly results from the distribution of the obstacles (Figure 8). In the Low Obstacle Density Area, the obstacle spacing is close to the body length. Thus, there are situations in which both monitors have obstacles displayed. When handling multiple obstacles on both sides, the human operator has to focus on both of the camera monitors, trying to go over obstacles on one side and avoid touching obstacles on the other side. To avoid both obstacles, stance legs (controlled by one finger) and swing legs (controlled by the other finger) must be coordinated. During long-distance walking while focusing on two monitors at the same time, the operator’s attention cannot always be highly concentrated. Inattention can lead to mistakes in judgment or operation, and increases the NOC in glove controlled locomotion. In contrast, in the High Obstacle Density Area, multiple obstacles only appear on the same side of the robot. Thus, the operator only needs to look at one camera monitor and focus on the control of the legs on that side. With reduced workload and less distraction, the operator can have more accurate control in obstacle avoidance.

Thus, as expected HHCI for tripod gaits is likely to be easiest to use when focusing on one leg’s placement at a time. Thus, for an application such as munitions response (SERDP, 2022) in which a robot might be exploring an area with infrequent objects of interest until the target object of interest is found, the autonomous gait might be used for much of the locomotion, and then as the robot gets closer the user can switch to HHCI. Once at the object of interest, the robot would be positioned such that rear leg placement is not as critical and operator can focus on how actions affect front legs.

In addition, more adjustments could be added. Potentially, more could be done in the computer visualization: rather than two videos stacked on top of each other, one view could be presented with both legs overlaid. The use of additional fingers for different legs, or switchable modes could improve performance.

Nonetheless, in all cases, HHCI is an improvement over fixed gaits, and would be a good candidate when autonomous terrain categorization is not available.

### 4.3 Future Interface Development

There are two major limitations to the gloves presented here: lack of steering and haptic feedback. These will be the basis of future work.

Steering is essential to controlling a robot in a three dimensional environment. Adduction/abduction at the MCP can be determined with additional sensors. Now we have demonstrated the TPM method in 2D, we can determine hip angle of the robot using the same approach. Alternatively, since frequent adduction and abduction movement can be uncomfortable, it would also be possible to use rotation at the user’s wrist to control steering direction. It is also possible to use a secondary joystick for steering, a method we have implemented in order to play a search-like game.

The present control interface only provides vision feedback to the operator without any haptic feedback. Visual feedback can be improved to manage attention following the principles of interaction efficiency (Goodrich and Olsen, 2003). Alternatively wearable Virtual Reality devices could be used. Finally, if haptic feedback is applied (Delft Haptics Lab, 1994; Barros et al., 2015; Hoshino et al., 2018; Huang et al., 2019; Protocom, 2022), the user may be able to “feel their way” through environments with limited vision or feel objects buried in sand.

Furthermore, future work can explore different ways to use all five fingers on the human hand. Because we showed that two fingers alone can control the two tripods of walking gait, we can envision switching between modes for walking (in which all legs move) and in-place motions (in which individual legs move, but stance legs stay planted). In the future, the ideal control interface may be a hybrid of manual and autonomous control, allowing the user to correct AI’s walking behavior in real time. Furthermore, the user’s inputs may be able to be compared with programmed gaits to enable gaits to adapt to user preferences.

Exploring these avenues will enable next steps in evaluation.

### 4.4 Future Evaluations

The NASA-TLX (Hart and Staveland, 1988) (Hart, 2006) is a standardized study to compare interfaces, which could be applied to compare our approach with other interface types, as shown in Figure 2. There are six dimensions (mental demand, physical demand, temporal demand, effort, performance and frustration level). Our hope is that because only finger motions are required, that the effort will be less and the mental demand will be comparable to that of using a joystick. For tasks such as placing a foot on a specific goal, we would expect less frustration, mental and temporal demands than scale model control. This can be evaluated with larger datasets for simulated and physical robots, where intervention is more likely to be required.

As human robot interfaces are being developed, wearable and intuitive smart devices can be important because they change the robot from a tool to be wielded to an extension of the user’s own body. This work shows that we can take advantage of similarity between human hand anatomy and robot design, to create a working interface. It is our hope that this will enable users without extensive robotics training to quickly learn to control robots as needed. In challenging and distracting environments, such as underwater or field work, lightweight one-hand interfaces are likely to be especially valuable.

## Data Availability

The original contributions presented in the study are included in the article/Supplementary Material, further inquiries can be directed to the corresponding author.
